# Grossly Erroneous Report of a Case in St. George's Hospital

**Published:** 1829-01-01

**Authors:** 


### FALSE REPORTS.

When Cullen observed, that in medicine there were more false facts than false theories, he little imagined that, in less than half a century from his decease, a regularly organized manufactory of the former would flourish in this country. The system of delusion at present carried on; the misrepresentations and falsehoods promulgated in the profession, are absolutely astonishing, and incredible to all but those who are behind the scenes. It is well known that, at present, the most extensive and valuable channel of information possessed by the profession is HOSPITAL REPORTING. If then, this stream of information be poisoned at its source, and its waters corrupted with misrepresentation and malevolence, the PUBLIC are drinking not a wholesome and salutary beverage, but rather, like Darius of old, are thirstily draining off a foul and a muddy draught!

We have often detected and exposed the ignorance and inaccuracy of the HOSPITAL REPORTS in the LANCET, and others have detected and exposed them also. Mr. Earle has publickly asserted, that not a single case of his, has been reported in that Journal with even an approach to correctness; and the same has been the case at St. George's Hospital. We heard a gentleman attending that institution, declare upon his honour, that the reports in the Lancet have often been absurd, not seldom *malâ fide* distorted, but always inaccurate. Its reporters are in general *en masque;* when known, universally shunned, except by the lowest of the low; and sometimes kicked out of the hospitals which they haunt, situations and circumstances highly favourable, no doubt, to the proper recording of facts!

An instance, one amongst a thousand, of the inaccuracy of these Lancet Reporters, has lately occurred at St. George's Hospital. The case was that of a man with a fracture of the skull, reported, on the same day in the Medical Gazette and the Lancet. Passing over the erroneous account of the treatment and dates in the latter, which are trifles compared to what follows, we come to the dissection. Let us compare the statements in these different Journals.

"On the post mortem examination, it was found that the fracture extended *to the left side of the head, as far as the petrous portion of the temporal bone,* and there was great extravasation of fluid under the scalp. The *dura mater was thickened,* and matter formed *on the surface of the brain,* and also between the bone and dura mater. THE THORACIC AND ABDOMINAL VISCERA WERE HEALTHY!"—*Lancet.*

"Much extravasation was found beneath the scalp, particularly in the occipital region. On raising the skull-cap, *the dura mater was found to be perfectly sound at the part where the trephine was applied.* In the direction of the spine, and transverse ridge of the occipital bone, where the fracture, as will presently be shewn, had extended, *a thin layer of blood was effused on the membrane,* which was greatly inflamed, indeed actually sloughy. Opposite this inflamed portion of the dura mater, *the tunica arachnoides was also inflamed, and covered with coagulable lymph.* There was some, but not very much fluid in the ventricles; no extravasation in, or rupture of the brain; little or no effusion of blood at the basis.

"The fracture extended from the spot where it was first discovered round the occipital bone, across the left branch of the lambdoid suture, obliquely over the petrous part of the left temporal bone, between the sella turcica and cuneiform process of the occipital bone; then over the right petrous portion to the place from which we started, completing the circle, and literally breaking off the back of the head. *Numerous and large depositions of lymph and pus existed in the liver, the texture of the viscus around being perceptibly engorged and inflamed. Similar appearances were discovered in the right lung—none in the left.* The spleen and other viscera were healthy; the urine was bilious."—MED. GAZ. No. 44.

Now it is obvious, that if the Gazette account of the dissection be right, the other must be totally wrong. The sins both of omission and commission on the part of the Reporter for the LANCET, are intolerably gross, and calculated to produce on the mind of his reader the most mischievous delusion.* He says that the viscera were healthy, though the liver was filled with purulent depôts, and the right lung disorganized in a similar manner!! There were present at the dissection, MR. BRODIE, DR. HEWETT, DR. JAMES JOHNSON, MR. PALMER of SUFFOLK PLACE, the HOUSE-SURGEONS, and a number of the PUPILS, all of whom can attest the truth of the account in the GAZETTE—the falsehood of that in the LANCET. There is no excuse whatever for the reporter of the latter, as the liver was examined, in the presence of all, and several remarks delivered by Mr. Brodie to the students on the frequency of hepatic abscess in injuries of the head! That the Lancet will deny it all, in the teeth of the testimony of those who were present, is probable enough, and consistent with the plan it has always pursued. The PUBLIC, however, are not to be hoodwinked.

---

* The Editor, who was present at the dissection, can confirm the accuracy of the report in the MEDICAL GAZETTE; but, *in this instance,* he absolves the reporter in the LANCET, from any wish to deceive. He appears to have given a highly erroneous statement of the case, from want of attention to what passed before his eyes, rather than from any intention to deceive. It will be seen whether the Journal which promulgated, will candidly correct the error, or endeavour to keep up the delusion.—ED.

